# Multimodal Non-Invasive Skin Imaging in Dermatology: Current Modalities, Clinical Applications, and Future Integration

**DOI:** 10.3390/diagnostics16142149

**Published:** 2026-07-09

**Authors:** Jin-Yi Deng, Jian-Ping Wei, Nafeixia Maimaitiyili, Xin-Yi Li, Qiu-Ting Zheng

**Affiliations:** 1 Non-Invasive Skin Imaging Center, Shenzhen Center for Chronic Disease Control, Shenzhen Dermatology Hospital, Shenzhen Institute of Dermatology, Shenzhen 518020, China13516534250@163.com (J.-P.W.); 2 Department of Medical Ultrasound, Shanghai Skin Disease Hospital, School of Medicine, Tongji University, Shanghai 200443, China

**Keywords:** multimodal non-invasive skin imaging, dermoscopy, optical coherence tomography, reflectance confocal microscopy, high-frequency ultrasound, skin imaging center

## Abstract

Multimodal non-invasive skin imaging combines complementary technologies, including dermoscopy, reflectance confocal microscopy (RCM), optical coherence tomography (OCT), line-field confocal optical coherence tomography (LC-OCT), and high-frequency ultrasound (HFUS), to enable comprehensive evaluation of skin lesions across multiple spatial scales. While dermoscopy facilitates assessment of superficial morphologic features, RCM provides near-cellular resolution imaging, C-OCT and LC-OCT offer high-resolution structural visualization at greater depths, and HFUS enables evaluation of deeper tissue architecture and lesion extent. The complementary strengths of these modalities support more accurate diagnosis, disease monitoring, and treatment assessment. This review summarizes the principles, clinical applications, advantages, and limitations of major non-invasive imaging modalities, with a particular emphasis on multimodal integration. We further propose a conceptual framework for a multimodal non-invasive skin imaging center, highlighting the roles of workflow standardization, multidisciplinary collaboration, and integrated data management. Current challenges, including implementation costs, data governance, and workforce training, are also discussed. Future developments in artificial intelligence, multicenter collaboration, and imaging-based treatment monitoring are expected to further advance precision dermatology and facilitate the broader clinical adoption of multimodal skin imaging.

## 1. Introduction

Non-invasive skin imaging has become an essential component of modern dermatology, enabling in vivo evaluation of skin lesions while overcoming many limitations associated with conventional histopathological examination. Over the past decade, dermoscopy, reflectance confocal microscopy (RCM), Optical coherence tomography (OCT), line-field confocal optical coherence tomography (LC-OCT), and high-frequency ultrasound (HFUS) have demonstrated substantial clinical utility in the diagnosis and management of skin tumors, inflammatory dermatoses, and aesthetic conditions. Among these modalities, LC-OCT has emerged as a key technological advance, serving as a bridge between conventional OCT and RCM by combining the near-cellular resolution of RCM with the greater imaging depth of OCT while providing real-time cross-sectional and en face imaging. However, the inherent limitations of these modalities in terms of imaging depth and resolution have driven increasing interest in multimodal imaging strategies. Beyond integrating complementary imaging information, a major challenge is translating these advantages into standardized clinical pathways and their systematic implementation through dedicated non-invasive imaging centers. Although several emerging non-invasive imaging techniques, such as optoacoustic mesoscopy and multispectral imaging, have shown promise in dermatologic research, their clinical adoption and evidence base remain limited. Accordingly, this review focuses on dermoscopy, RCM, OCT/LC-OCT, and HFUS, which currently have the broadest clinical uptake and the strongest evidence base (see Table 1). It summarizes their underlying principles, clinical applications, strengths, and limitations while highlighting their complementary roles in multimodal skin imaging. We further present a conceptual framework for the potential organization of a non-invasive skin imaging center, including considerations related to workflow integration, multidisciplinary collaboration, and data management. In addition, we discuss current challenges and future research directions. The present review aims to provide a comprehensive reference for the clinical application and continued development of non-invasive skin imaging, while also offering a conceptual framework to guide the future establishment of non-invasive skin imaging centers.

This article is a narrative review rather than a systematic review. Relevant literature was identified through searches of PubMed, Web of Science, and Google Scholar using combinations of keywords including “dermoscopy”, “reflectance confocal microscopy”, “Optical Coherence Tomography”, “high-frequency ultrasound”, “multimodal imaging”, “non-invasive skin imaging”, and “dermatology”. Additional relevant publications were identified through manual screening of reference lists. Priority was given to recent studies, systematic reviews, meta-analyses, clinical investigations, and articles considered influential in the development of multimodal skin imaging.

## 2. Dermoscopy

Dermoscopy uses epidermal translucency and polarized light to enhance visualization of surface and subsurface structures of pigmented skin lesions at magnifications ranging from 10× to 400× [1,2].

Dermoscopy has become an indispensable tool in the evaluation of a wide spectrum of benign and malignant skin neoplasms. In basal cell carcinoma (BCC), characteristic findings such as arborizing vessels, blue-gray ovoid nests, leaf-like areas, and shiny white structures improve diagnostic accuracy and aid subtype recognition [3]. In melanoma, dermoscopy enables the differentiation of histological subtypes, which is critical for selecting appropriate management strategies [4]. Non-contact dermoscopy is particularly valuable for vascular assessment and, when combined with clinical evaluation, contributes significantly to the diagnosis of inflammatory dermatoses [5]. In hair and scalp disorders, dermoscopy, also known as trichoscopy, enables the assessment of hair signs, vascular patterns, pigment patterns, and interfollicular patterns [6]. A representative example is androgenetic alopecia, in which trichoscopy reveals characteristic features such as hair diameter diversity, increased vellus hairs, and the peripilar sign, thereby aiding diagnosis and disease assessment [7]. Dermoscopy has also proven useful in the diagnosis of infectious and parasitic diseases. In scabies, the characteristic “jet with contrail” sign represents the anterior portion of the mite within the burrow [8]. In pediculosis, dermoscopy allows reliable identification of lice, viable nits, and empty eggshells, thereby aiding both diagnosis and assessment of treatment response [9]. In aesthetic medicine, dermoscopy has also been applied to the evaluation of skin photoaging [10], melasma [11], and treatment outcomes following laser therapy and other cosmetic procedures [12].

Digital dermatoscopy is the combination of normal dermatoscopes with digital high-magnification cameras. Digital dermoscopy systems enable standardized image acquisition, storage, sequential monitoring, and automated analysis of skin lesions, facilitating the detection of subtle morphological changes over time [13]. These systems are particularly valuable for monitoring patients at high risk for melanoma and for supporting teledermatology and artificial intelligence-assisted diagnostic workflows [14].

Despite its clinical utility, dermoscopy has several inherent limitations in the evaluation of skin diseases. Its imaging depth is restricted to approximately 200–300 μm, allowing visualization primarily of superficial epidermal and dermo-epidermal structures while providing little information about deeper tissues [15]. Certain lesions may also exhibit overlapping dermoscopic features, posing diagnostic challenges. For example, trichoblastoma, a benign adnexal neoplasm, can closely mimic BCC both clinically and dermoscopically [16]. Furthermore, the diagnostic performance of dermoscopy remains highly dependent on the examiner’s expertise and experience [17].

Evidence suggests that integrating dermoscopy with other non-invasive imaging modalities can overcome some of these limitations. In a retrospective study, Witkowski et al. reported that, in the diagnosis of pink basal cell carcinoma, dermoscopy alone showed lower specificity and positive predictive value (PPV) than either RCM alone or the combined use of dermoscopy and RCM, and was associated with diagnostic errors [18]. While dermoscopy substantially improves the assessment of skin lesions compared with unaided clinical examination, the addition of cellular-level information provided by RCM can further enhance diagnostic accuracy and confidence. These findings highlight the value of multimodal non-invasive imaging approaches in improving diagnostic performance and supporting clinical decision-making.

## 3. Reflectance Confocal Microscopy

Reflectance confocal microscopy (RCM) is a non-invasive imaging modality that provides real-time, in vivo visualization of the skin at near-histological resolution. By detecting differences in the refractive indices and backscattering properties of tissue components, RCM achieves an axial resolution of 3–5 μm, a lateral resolution of approximately 1 μm, and an imaging depth of up to 250 μm [19], enabling cellular-level evaluation of the epidermis and papillary dermis. This capability allows detailed morphological assessment of skin lesions and may reduce the need for unnecessary biopsies of benign lesions [20].

RCM has demonstrated broad clinical utility for diagnosing and monitoring both neoplastic and inflammatory skin diseases. In skin oncology, RCM provides cellular-level morphological information [21] that facilitates differentiation between benign and malignant lesions. Its high sensitivity for melanin detection makes it particularly valuable for evaluating pigmentary lesions, including diagnostically challenging hypomelanotic melanomas [22]. It also shows high sensitivity and specificity for the diagnosis of non-melanoma skin cancers, such as BCC [23]. Beyond tumor assessment, RCM enables non-invasive monitoring of inflammatory dermatoses by quantifying dynamic cellular and vascular changes, with studies demonstrating its utility in evaluating treatment responses in psoriasis and in monitoring biologic therapy [24,25,26]. Furthermore, the development of handheld RCM (HH-RCM) has improved accessibility to anatomically challenging sites, thereby expanding its clinical applications [23].

RCM has also emerged as a valuable tool in aesthetic dermatology, enabling the non-invasive assessment of skin aging, pigmentary changes, and treatment responses to cosmetic procedures, including laser resurfacing and anti-aging interventions [27].

Despite its near-histological resolution, RCM is inherently limited by its shallow imaging depth, which is generally restricted to the papillary dermis or superficial reticular dermis [28]. As a result, deeper dermal structures and deep surgical margins cannot be reliably assessed. Janowska et al. noted that HFUS, unlike RCM, can assess both lateral and deep lesion margins, underscoring the complementary roles of these two imaging modalities [29]. In addition, the relatively small field of view, lengthy examination times, high equipment and maintenance costs, and the need for specialized training remain important barriers to the broader implementation of RCM in routine clinical practice.

## 4. Optical Coherence Tomography

Optical coherence tomography (OCT) is a non-invasive imaging technique based on low-coherence interferometry that generates real-time cross-sectional images of biological tissues. Conventional OCT (C-OCT) systems typically provide an axial resolution of approximately 5 μm, a lateral resolution of approximately 7.5 μm, and a penetration depth of up to 2 mm, enabling visualization of the epidermis, dermoepidermal junction, and superficial dermis [30].

To date, the most extensively investigated application of OCT in dermatology has been the diagnosis and management of non-melanoma skin cancer, particularly basal cell carcinoma (BCC). Characteristic OCT features of BCC include hyporeflective tumor islands, alteration of the DEJ, well-circumscribed signal-poor areas, and dilated vessels [31]. Several studies have demonstrated that OCT provides high concordance with histopathology and can accurately delineate BCC margins preoperatively, improving surgical planning, facilitating Mohs micrographic surgery, and reducing unnecessary tissue excision [32]. In addition to skin cancer, OCT has also shown value in nail unit imaging. By enabling non-invasive visualization of the nail plate, nail bed, and periungual structures, OCT can provide complementary structural information in disorders such as nail psoriasis and onychomycosis [33].

Recent technological advances have led to the development of line-field confocal optical coherence tomography (LC-OCT), one of the most significant innovations in non-invasive skin imaging in the past decade. LC-OCT combines the optical principles of OCT and RCM through line-field illumination [34], effectively bridging the resolution–depth gap between the two modalities. The technique provides in vivo and real-time images with 1.2 μm axial resolution/500 μm penetration depth/1.3 mm lateral field [35].

LC-OCT has demonstrated broad clinical utility in the evaluation of skin tumors and a variety of inflammatory and infectious dermatoses. A recent systematic review and meta-analysis reported excellent diagnostic accuracy for malignant skin tumors (AUC = 0.914), with superior sensitivity, specificity, positive predictive value, and negative predictive value compared with dermoscopy, highlighting its growing role in dermatologic diagnosis [36]. Additionally, LC-OCT has been increasingly applied to inflammatory and infectious skin diseases. The technique allows real-time assessment of architectural features such as epidermal thickness, fluid accumulation, DEJ morphology, vascular architecture, inflammatory infiltrates, and foreign bodies [37].

In addition, LC-OCT combined with automated three-dimensional (3D) segmentation enables quantitative assessment of dermal fiber aging and objective monitoring of anti-aging skincare efficacy [38]. LC-OCT can also non-invasively visualize hyaluronic acid dermal fillers in real time, facilitating precise localization of filler deposits and longitudinal monitoring of their distribution, which can help optimize injection procedures and manage filler-related complications [39].

Despite these advantages, conventional OCT lacks sufficient lateral resolution to resolve individual cells, limiting its ability to provide true histology-like diagnosis. Although LC-OCT substantially improves image resolution, its penetration depth is limited to approximately 500 μm, restricting evaluation of the deep dermis and subcutaneous tissue, including deep tumor margins and deeply placed fillers.

These limitations highlight the complementary role of OCT/LC-OCT with other non-invasive imaging modalities. For example, in the diagnosis of hidrocystomas, LC-OCT provides high-resolution visualization of superficial cystic structures, whereas ultra-high-frequency ultrasound (UHFUS) offers greater imaging depth [40], enabling assessment of deep lesion margins and overall anatomical architecture.

## 5. High-Frequency Ultrasound

High-frequency ultrasound (HFUS), typically defined as ultrasound operating at frequencies above 15 MHz, generates images by reflecting high-frequency sound waves and detecting differences in tissue acoustic impedance, enabling visualization of deeper skin structures and lesion extent [41].

HFUS provides an imaging depth of up to 10 mm [15] and can be integrated with advanced ultrasound techniques to enhance diagnostic performance. Among these, shear-wave elastography (SWE) enables quantitative assessment of tissue stiffness and has become one of the most promising ultrasound-based approaches for evaluating skin fibrosis. In systemic sclerosis (SSc), SWE has demonstrated excellent reproducibility and diagnostic accuracy, with reported intraclass correlation coefficients reaching 0.987 compared with 0.941 for the modified Rodnan skin score (mRSS), as well as sensitivity and specificity exceeding 0.90 and 0.93, respectively, for distinguishing patients from healthy controls [42]. Beyond fibrosis assessment, combining SWE with superb microvascular imaging (SMI) improves the differentiation of benign and malignant skin tumors [43], while combining SWE with angio planewave ultrasensitive imaging (AP) facilitates comprehensive evaluation and individualized management of keloids [44]. As a radiation-free modality that permits repeated examinations, HFUS has been widely applied in tumor depth assessment [45,46], localization of subcutaneous lesions [47,48], and evaluation of inflammatory disease severity [49,50]. In aesthetic dermatology, HFUS can accurately identify and characterize various periorbital filler types, aiding in the detection of complications such as infection and calcification [51,52]. In addition, high-intensity focused ultrasound (HIFU), a therapeutic ultrasound technology, promotes tissue remodeling through controlled thermal and mechanical effects, making it an important tool for facial rejuvenation [53].

However, ultrasound has limited resolution and cannot display color or textural details. Wang et al. reported that HFUS cannot reliably detect lesions thinner than 0.1 mm [54]. In addition, HFUS cannot provide the cellular-level information required for histopathological assessment, and current evidence remains insufficient to support its use in distinguishing melanoma in situ from benign melanocytic nevi [55]. Other limitations include operator dependence and inter-session variability in measurements [56].

These limitations may be mitigated through multimodal imaging approaches. In a prospective study of 12 patients with basal cell carcinoma, Navarrete-Dechent et al. demonstrated that combining dermoscopy, HFUS, and RCM increased the detection of BCC-specific features from 17% with HFUS alone to 100% with multimodal assessment [57].

## 6. Integration and Collaboration

### 6.1. Multimodal Imaging Integration

The evidence reviewed above highlights that, despite their distinct strengths, dermoscopy, RCM, OCT, and HFUS are each limited by their imaging capabilities. Consequently, increasing attention has been directed toward multimodal non-invasive skin imaging. By combining modalities with complementary depths and resolutions, multimodal imaging enables comprehensive assessment across skin layers and spatial scales, from the epidermal surface to subcutaneous tissues and from macroscopic architecture to cellular-level morphology [58,59], thereby promoting more standardized diagnostic approaches [60].

Against this background, integrating multiple imaging modalities into standardized and reproducible diagnostic workflows, rather than simply combining individual techniques, has become an important focus of current research. To date, reports describing fully integrated multimodal non-invasive skin imaging centers remain scarce, and standardized implementation models have not yet been established. Nevertheless, the growing body of evidence supporting multimodal imaging integration suggests a need for more coordinated diagnostic infrastructures. Therefore, the following sections outline a conceptual framework for a multimodal non-invasive skin imaging center, representing a potential future model for integrating imaging technologies, data management, multidisciplinary collaboration, and clinical decision-making, rather than a description of current routine clinical practice. The potential benefits of the proposed multimodal non-invasive skin imaging center for different stakeholder groups are also discussed (see Table 2).

### 6.2. A Conceptual Framework for the Non-Invasive Skin Imaging Center

Given the limited real-world experience with fully integrated multimodal imaging centers in dermatology, we propose a conceptual framework that may guide future development. In this proposed model, the design of a non-invasive skin imaging center would be guided by two key principles: patient-centeredness and technology integration. Patient-centeredness aims to optimize examination pathways and the care environment, allowing patients to complete multiple imaging examinations during a single visit. Technology integration emphasizes the coordinated use of complementary imaging devices, interoperable data systems [61], and multimodal image management platforms that facilitate efficient workflows and interdisciplinary communication.

These principles are intended to support three major objectives. First, multimodal data fusion could be facilitated via an integrated imaging data management platform [62] that creates a unified disease-specific database. Second, workflow standardization could be promoted through standard operating procedures (SOPs) covering image acquisition, quality control, and report generation [61]. Third, multidisciplinary collaboration could be facilitated through a cross-disciplinary platform that integrates imaging, clinical, and pathological data [63].

The proposed imaging center may also play a role in aesthetic dermatology by enabling objective evaluation of cosmetic procedures and treatment outcomes [12].

### 6.3. Multidisciplinary Team

In this proposed framework, a multimodal non-invasive skin imaging center could be supported [64] by a multidisciplinary team (MDT) comprising dermatologists, sonologists, skin imaging technicians, pathologists, radiologists, and algorithm engineers.

A potential collaborative workflow may comprise three stages. During pre-imaging consultation, dermatologists and sonologists jointly assess complex cases, consulting pathologists when necessary to determine the optimal imaging strategy. Image interpretation follows a two-tier reading system: for all modalities except ultrasound, technicians perform preprocessing and quality assessment before dermatologists review the images; ultrasound examinations are performed and interpreted by sonologists, with dermatologists involved as needed. Complex or inconclusive cases undergo multidisciplinary review based on integrated imaging, clinical, and pathological findings. Finally, follow-up and treatment decisions, including biopsy, surgery, and non-invasive interventions, could be guided by multimodal imaging findings. The proportion of treatment plans modified by these findings may be monitored as a quality indicator.

### 6.4. Workflow Optimization

In the proposed model, workflow optimization encompasses both patient-level diagnostic pathways and organizational integration within the non-invasive skin imaging center. For selected patient populations, particularly those undergoing surveillance for melanoma or other high-risk skin lesions, a triage layer may precede multimodal imaging assessment. Digital monitoring tools such as total body photography (TBP) and sequential digital dermoscopy imaging (SDDI) can be used to identify suspicious or evolving lesions and prioritize them for further evaluation. Following this initial risk stratification step, selected lesions may undergo multimodal assessment using dermoscopy, RCM, OCT, HFUS, and other complementary imaging modalities.

At the organizational level, the integration of these imaging resources within a single diagnostic unit could enable multiple examinations during a single visit. Combined with standardized reporting systems, integrated data management, and multidisciplinary collaboration, this model has the potential to streamline clinical workflows, improve patient experience, promote interdisciplinary collaboration, and support more efficient clinical decision-making. These anticipated benefits remain to be validated in future real-world studies.

## 7. Challenges

Multimodal non-invasive skin imaging marks an important step toward the digitalization and intelligent transformation of dermatologic practice. By integrating advanced imaging technologies, it facilitates more accurate and efficient diagnoses. However, several challenges remain to be overcome.

First, the high costs of equipment and software development limit implementation. Multimodal imaging platforms require substantial investment in technologies such as HFUS, RCM, OCT, and data management systems. A phased implementation strategy, supported by research funding and industry–academia partnerships, may help reduce financial burden.

Second, data security and patient privacy are critical concerns. The large volume of sensitive data generated requires robust safeguards, including regulatory compliance, data de-identification, role-based access control, and regular security audits.

Third, multimodal image interpretation demands specialized expertise. Effective implementation of multimodal imaging requires clinicians to develop familiarity with the principles, interpretation, and limitations of multiple imaging modalities [65]. Structured training, mentorship, and multidisciplinary case discussions may enhance diagnostic competence and promote the routine use of multimodal imaging.

## 8. Future Perspectives

Recent advances in artificial intelligence (AI) have created new opportunities for multimodal skin imaging [66]. Although AI-assisted analysis has demonstrated encouraging results in individual imaging modalities, including RCM, LC-OCT, and HFUS [67], AI-driven multimodal integration remains an evolving objective rather than an established clinical practice. Future research should focus on validating these multimodal algorithms across diverse patient populations and clinical settings, as well as improving the interpretability and generalizability of AI-assisted decision-support systems.

Another important priority is the establishment of multicenter collaborative networks to facilitate large-scale validation of multimodal imaging workflows. Standardized imaging protocols, reporting criteria, and shared image databases will be essential for improving reproducibility and enabling meaningful comparisons across studies. Such efforts may also support the development of consensus guidelines and accelerate the integration of multimodal imaging into routine clinical practice.

In addition to diagnosis, multimodal imaging is increasingly being explored for treatment monitoring and longitudinal follow-up. Recent studies have demonstrated that LC-OCT and RCM can accurately detect residual tumor structures and distinguish disease recurrence from radiation-induced tissue changes in patients with non-melanoma skin cancer following radiotherapy [68]. These findings highlight the potential of multimodal imaging as a non-invasive approach for treatment–response assessment, recurrence surveillance, and personalized follow-up strategies. As evidence continues to accumulate, treatment monitoring may become an increasingly important component of integrated skin imaging platforms.

## Figures and Tables

**Table 1:** Functions and Typical Application Scenarios of Dermoscopy, RCM, OCT, and HFUS.

Device Type	Key Specifications	Primary Function	Advantages	Limitations	Typical Applications
**Dermoscopy**	• Imaging depth: ~0.2 mm • Magnification: 10×–400×	• Observation of structures from the epidermis to the superficial dermis • Differentiation of pigmentation disorders • Analysis of hair and scalp disorders	• Optical imaging, high speed • Real-time imaging • 10×–400× magnification • Non-invasive, easy to use, low cost	• Limited penetration depth • Dependent on the physician’s experience • Cannot quantify the elastic modulus	• Initial screening of nevi/melanoma • Diagnosis of alopecia areata/androgenetic alopecia • Detection of tinea capitis and nits
**RCM**	• Imaging depth: ~0.25 mm • Resolution (L/A): 1/3–5 μm • FOV: 8 × 8 mm	• Cellular-level imaging • Three-dimensional skin structure reconstruction • Dynamic monitoring of inflammatory/tumor cells	• Provides cellular-level (histology-like) detail • Non-invasive alternative to some biopsies • Capable of observing melanocyte distribution	• Small scanning area • Lengthy examination time • High maintenance cost	• Screening for suspected basal cell carcinoma • Assessment of vitiligo progression • Observation of microscopic inflammation in psoriasis
**C-OCT**	• Imaging depth: up to 2 mm • Resolution (L/A): 7.5/5 μm	• Real-time cross-sectional imaging • Visualization of epidermis, DEJ, and superficial dermis • Structural assessment of skin and nail units	• Non-invasive, real-time cross-sectional imaging • Good penetration • High concordance with histopathology	• Insufficient cellular resolution • Unable to provide histology-like images • Limited characterization of individual cells	• Diagnosis and margin assessment of BCC • Preoperative surgical planning • Nail psoriasis and onychomycosis evaluation
**LC-OCT**	• Imaging depth: ~0.5 mm • Resolution (L/A): 1.3/1.2 μm	• High-resolution in vivo skin imaging • Combined OCT–RCM imaging approach • Assessment of epidermal and superficial dermal architecture	• Bridges the resolution–depth gap between OCT and RCM • Near histology-like resolution • Real-time imaging with high diagnostic accuracy	• Limited penetration depth • Relatively small lateral field • Restricted evaluation of deep dermis and subcutaneous tissue	• Skin tumors (malignant and benign) • Inflammatory and infectious dermatoses • Dermal filler localization and treatment monitoring
**HFUS**	• Imaging depth: up to 10 mm • Frequency: 20–100 MHz	• Stratified imaging of epidermis, dermis, and subcutaneous tissue • Tumor depth assessment • Aesthetic evaluations	• Greatest penetration depth • Supports multiple imaging modes • Radiation-free, repeatable testing	• Inability to display color or texture details • Requires operator training	• Localization of subcutaneous masses • Assessment of inflammatory severity • Prediction of scar hyperplasia risk • Tracking of filler migration

**Table 2:** Potential Benefits of the Proposed Multimodal Non-invasive Skin Imaging Center.

Beneficiary Group	Potential Benefits
**Patients**	Non-invasive and radiation-free imaging that may reduce the need for invasive diagnostic procedures in selected patients. Potential for earlier detection of subtle or clinically occult lesions, which may support timely diagnosis and treatment. Real-time treatment assessment that may facilitate patient communication and longitudinal follow-up. Potential for more precise diagnoses, which may reduce unnecessary investigations or treatments and improve clinical efficiency.
**Physicians**	Potential to support more standardized diagnostic workflows and clinical decision-making. Potential to improve diagnostic confidence through multimodal assessment of complex skin lesions. May contribute to improved patient experience through more comprehensive diagnostic evaluation.
**Clinical Department**	Potential to expand diagnostic capabilities for complex dermatologic conditions. May facilitate imaging data collection for clinical research and education. Potential to support workflow standardization and interdisciplinary collaboration.
**Hospital**	Potential to support the development of specialized dermatologic imaging services. May reduce reliance on invasive diagnostic procedures in selected clinical scenarios. Potential to improve care coordination and diagnostic efficiency through integrated imaging workflows. May facilitate the adoption of precision dermatology and digital healthcare strategies.

## Data Availability

No new data were created or analyzed in this study. Data sharing is not applicable to this article.
